# The Population Genetics of *Pseudomonas aeruginosa* Isolates from Different Patient Populations Exhibits High-Level Host Specificity

**DOI:** 10.1371/journal.pone.0013482

**Published:** 2010-10-19

**Authors:** Rosa van Mansfeld, Irene Jongerden, Martin Bootsma, Anton Buiting, Marc Bonten, Rob Willems

**Affiliations:** 1 Department of Medical Microbiology, University Medical Centre Utrecht, Utrecht, The Netherlands; 2 Department of Intensive Care Medicine, University Medical Centre Utrecht, Utrecht, The Netherlands; 3 Julius Center for Health Research and Primary Care, University Medical Centre Utrecht, and Department of Mathematics, Faculty of Science, Utrecht University, Utrecht, The Netherlands; 4 Department of Medical Microbiology and Immunology, St Elisabeth Hospital, Tilburg, The Netherlands; Universidade Federal de Minas Gerais, Brazil

## Abstract

**Objective:**

To determine whether highly prevalent *P. aeruginosa* sequence types (ST) in Dutch cystic fibrosis (CF) patients are specifically linked to CF patients we investigated the population structure of *P. aeruginosa* from different clinical backgrounds. We first selected the optimal genotyping method by comparing pulsed-field gel electrophoresis (PFGE), multilocus sequence typing (MLST) and multilocus variable number tandem-repeat analysis (MLVA).

**Methods:**

Selected *P. aeruginosa* isolates (n = 60) were genotyped with PFGE, MLST and MLVA to determine the diversity index (DI) and congruence (adjusted Rand and Wallace coefficients). Subsequently, isolates from patients admitted to two different ICUs (n = 205), from CF patients (n = 100) and from non-ICU, non-CF patients (n = 58, of which 19 were community acquired) were genotyped with MLVA to determine distribution of genotypes and genetic diversity.

**Results:**

Congruence between the typing methods was >79% and DIs were similar and all >0.963. Based on costs, ease, speed and possibilities to compare results between labs an adapted MLVA scheme called MLVA9-Utrecht was selected as the preferred typing method. In 363 clinical isolates 252 different MLVA types (MTs) were identified, indicating a highly diverse population (DI  = 0.995; CI  = 0.993–0.997). DI levels were similarly high in the diverse clinical sources (all >0.981) and only eight genotypes were shared. MTs were highly specific (>80%) for the different patient populations, even for similar patient groups (ICU patients) in two distinct geographic regions, with only three of 142 ICU genotypes detected in both ICUs. The two major CF clones were unique to CF patients.

**Conclusion:**

The population structure of *P. aeruginosa* isolates is highly diverse and population specific without evidence for a core lineage in which major CF, hospital or community clones co-cluster. The two genotypes highly prevalent among Dutch CF patients appeared unique to CF patients, suggesting specific adaptation of these clones to the CF lung.

## Introduction

*Pseudomonas aeruginosa* can cause nosocomial infections in immuno-compromised patients and patients in intensive care units (ICUs), and is a major cause of morbidity and mortality in patients with cystic fibrosis (CF) [1]. Molecular typing studies revealed the presence of so-called epidemic strains, frequently transmitted between CF patients and associated with higher morbidity and mortality [2], [3], [4], [5], [6]. As a consequence, many countries implemented segregation policies for CF patients [7].

In a previous cross-sectional study, investigating the population structure of respiratory *P. aeruginosa* isolates among Dutch CF patients by using multilocus sequence typing (MLST), we described two sequence types (ST), ST406 and ST497 in 15% and 5% of all patients infected with *P. aeruginosa*, respectively [8]. Both STs were not genetically linked to previously described international epidemic clones, which were not detected in this CF population.

In order to determine whether these prevalent STs are specifically linked to patients with CF, or ubiquitously present in other patient populations, we aimed to investigate the genetic relatedness and population structure of *P. aeruginosa* isolates from CF and non-CF patients. To do so, a highly discriminatory, cheap and easy to perform typing scheme, which also allows results to be easily compared with international databases, is required. Pulsed-field gel electrophoresis (PFGE) has been the most widely used typing method, but does not allow easy comparison of results of different origin because of a relatively high degree of inter-performer variation and lack of an international comparative database. MLST provides sequence-based, and thus unambiguous, results, but is rather expensive. We, therefore, first determined whether multi-locus variable number tandem-repeat analysis (MLVA) could fulfill these criteria required for library typing, by comparing a new MLVA scheme, adjusted from the published *P. aeruginosa* MLVA scheme [9], to PFGE and MLST. After identifying the optimal typing scheme, based on discriminatory power, typeability, time, ease of interpretation and of international comparability and costs, we determined the population structure of multiple *P. aeruginosa* isolates from different epidemiological backgrounds.

## Materials and Methods

### Genotyping

To determine the optimal molecular typing method, 60 *P. aeruginosa* isolates from sputum or throat swab cultures obtained from 58 different CF patients visiting the University Medical Centre Utrecht (UMCU) in 2007, were typed by PFGE, MLST and MLVA9-Utrecht. This selection represented the genotypes and genetic diversity found in the Dutch CF patients as shown in a previous cross-sectional typing study. The Discriminatory Indices (DI) and the 95% confidence intervals (CI) were calculated as described before [10], [11] using Bionumerics 5.1(Applied Maths, St-Martens-Latem, Belgium). Criteria to assign isolates to clonal clusters (CCs) were defined as follows: PFGE types (PT) >80% similarity in band patters, MLVA types (MTs) with identical number of repeats in 8 out of 9 loci (single locus variants) and MLST types (STs) with identical sequence in 6 out of 7 loci. CCs were named after their presumed founder MT/ST, based on eBURST criteria [12]. The quantitative level of congruence between typing methods was calculated using the adjusted Rand and Wallace coefficients, available at http://www.comparingpartitions.info/. The adjusted Rand coefficient quantifies the global agreement between two methods, whereas the Wallace coefficient indicates the probability that two isolates classified as the same type by one method are also classified as the same type by another method [13]. MLVA9-Utrecht profiles were clustered with Bionumerics software (version 5.1) by using a categorical coefficient and a graphing method called minimum spanning tree [14].

### PFGE

For PFGE, 2% agarose plugs were made with equal volume bacterial suspension of 3 McFarland. Plugs were incubated overnight with 0.5 mg/ml lysozyme (Sigma-Aldrich, Zwijndrecht, Netherlands) at 37°C. Next 1 mg/ml proteinase K (VWR, International, Amsterdam, Netherlands) was added and plugs were incubated overnight at 56°C.

Plugs were washed for 30 min at 37°C once with 10 mM tris/1 mM EDTA (TE) buffer, then 0.75 mM phenyl-methyl-sulfonyl-fluoride (PMSF) in TE buffer, and again with TE buffer. Plugs were digested with *Spe*I 5 µl (50 U) in 25 µl NE buffer2 (Westburg, Leusden, Netherlands) and 220 µl water overnight at 37°C. Electrophoresis was performed with 1% agarosegel for 20 h at 6 V/cm with initial switch of 5.8 s and final switch of 38 s. *P. aeruginosa* strain ATCC 27853 was used as reference at minimal 5 lanes in each gel. The gels were stained with ethidiumbromide and bands were analysed with Bionumerics 5.1 (Applied Maths, St-Martens-Latem, Belgium). The band patterns were compared using the Dice-coefficient by using the unweighted pair group method to determine band similarity. Band patterns that were more than 80% identical were considered related conform the Tenover criteria [15], [16], which state that a 2–3 band difference indicates related strains. On average we observed 16 bands in our *P. aeruginosa* PFGE gels, resulting in the 80% cut-off. Typeability was defined as all isolates that produced a band pattern divided by all isolates tested.

### MLST and MLVA

Isolates were taken from the freezer and cultured on Trypticase Soy Agar II +5% sheep blood plates (Becton, The Netherlands) overnight at 37°C. A loop (few colonies) of bacterial cells were suspended in 20 µl lysis buffer (0.25% SDS, 0.05 M NaOH) and incubated at 95°C for 20 min. The cell lysate was spun by short centrifugation and diluted with 180 µl buffer (10 mM Tris-HCl, pH 8.5). After thoroughly mixing, another centrifugation for 5 min at 16,000×g was performed to remove cell debris. Supernatants were frozen at −20°C until further use. Two and a half µl of the lysate was used in the PCR reactions for MLST and MLVA.

For MLST a touchdown PCR was performed as described before, adapted from the protocol by Curran *et al* [17] with HotStarTaq Mastermix (Qiagen, Valencia, CA,USA). PCR products were sequenced (BaseClear, Leiden, the Netherlands) with the same primers as used for amplification. Sequences were analyzed using Bionumerics 5.1 (Applied Maths, St-Martens-Latem, Belgium).

Sequence types (STs) were compared to the *P. aeruginosa* Multilocus Sequence Typing website (http://pubmlst.org/paeruginosa/) developed by Keith Jolley [18] and new alleles and profiles were sent to the curator A. Baldwin.

For MLVA typing a touchdown PCR was performed adapted from the protocol by Vu-Thien *et al* adding Q-buffer (Qiagen Benelux B.V., Venlo, the Netherlands) using the published primers for the following variable-number-of-tandem-repeats (VNTRs): ms77, ms127, ms142, ms211, ms213, ms215, ms216, ms217 and ms223 (called MLVA9-Utrecht). The PCR was conducted as follows: 10 min at 96°C, then 10 cycles of 30 s at 95°C, 30 s at 65°C with 1°C less every cycle and 1 min at 72°C. This was followed by 25 cycles of 30 s at 95°C, 30 s at 55°C and 1 min at 72°C, and a final incubation of 10 min at 72°C followed. PCR products were separated on a 2% agarose gel by electrophoresis next to 100 bp DNA ladder (Invitrogen, The Netherlands). The size of each amplicon was measured using Bionumerics 5.1 (Applied Maths, St-Martens-Latem, Belgium) and the number of repeats was deduced by using the MLVA alleles assignment table on the *Pseudomonas aeruginosa* genotyping site (http://minisatellites.u-psud.fr/MLVAnet/). PA01 (ATCC BAA47) was used as control for checking consistency of allele assignments. Loci that repetitively did not yield a PCR product were assigned allele “99” to be able to include these isolates in subsequent cluster analysis. MLVA9-Utrecht types (MTs) were compared with the international database “pseudomonas2007” created by Gilles (http://minisatellites.u-psud.fr/MLVAnet/). Typeability was defined as the number of isolates for which repeat numbers could be inferred for all 9 loci divided by all isolates tested.

### Patients and bacterial isolates

To determine the *P. aeruginosa* population structure, 363 isolates were collected from four different patient populations: 100 respiratory isolates from 90 CF patients who either were cultured because of an exacerbation or screened for their annual check-up (group I) and 205 *P. aeruginosa* isolates from aspirate, sputum or throat swab screening cultures from patients admitted to intensive care units (ICU) (one isolate per type per patient) in two hospitals in the Netherlands (126 isolates from 97 patients in hospital 1 (group IIa) and 79 isolates from 64 patients in hospital 2 (group IIb). Screening cultures were executed on admission, twice weekly thereafter and on discharge during a period of 14 months in both hospitals. Hospital 1 is a tertiary referral (university) hospital and patients were included in two ICUs (10 and 8 beds, of which 6 and 7 beds on a ward, respectively) harboring a mixed adult patient population. Hospital 2 is non-university teaching hospital, located 80 kilometers from hospital 1, and here patients from two ICUs (8 and 8 beds, single rooms) also harboring a mixed adult patient population were included. In both ICUs, CF patients were excluded. In total, 1200 patients were admitted for more than 24 hours and screened (cultures were not available of 113 patients). Isolates of 161 of 194 colonized patients were typed and included in this study. Group III consisted of 39 non-respiratory clinical isolates from 38 non-CF patients and non-ICU patients admitted to hospital 1. These 38 patients were mostly long-stay patients (admitted > one month) in different wards, including surgery, neurology, oncology and internal medicine.

Group IV consisted of 19 isolates from 19 non-CF and non-ICU patients obtained within 48 hours after admission or during out-patient clinic visits at hospital 1. These isolates are considered “community acquired”. The community acquired isolates were cultured mainly from eyes, ears, wounds and screening cultures of patients admitted for stem cell transplantation. The ethical committees (METC) of both the University Medical Center Utrecht and the St Elisabeth Hospital Tilburg approved this study and waived the requirement for informed consent (METC Utrecht protocol number: 05/311, METC Tilburg protocol number: 0655), since cultures were obtained as part of the hospital surveillance program or clinical practice.

### Calculation of expected DI and MT distribution

The median value and the 95% confidence intervals for the DIs and the overlap in types between different clinical sources was calculated using Mathematica 7.0.1.0, (Wolfram Research, Champaign, Ill), by distributing the isolates 100,000 times, randomly, over the different clinical sources under the assumption that genotypes do not cluster. The number of isolates per group and the prevalence of the different MTs were considered fixed and only the distribution over the different groups was randomized.

## Results

### Adjusted MLVA9-Utrecht scheme

We first adjusted the published *P. aeruginosa* MLVA scheme, as originally described by Vu-Thien *et al*. The original scheme contained 15 variable number tandem-repeat (VNTR) loci, of which some, due to small repeat sizes, required analysis on a DNA sequencer. To create a robust MLVA scheme that was easy to perform without the need for a DNA sequencer, we tested different combinations of the original 15 VNTR loci and calculated the DIs for the different combinations in a set of 101 *P. aeruginosa* isolates (the 100 selected CF isolates plus PA01; ATCC BAA47). VNTR loci that were not selected in the final scheme were loci with too small repeat size (<15 nt) and loci that could not be amplified in >10% of the isolates. Based on these criteria we selected a subset of nine MLVA loci, ms77, ms127, ms142, ms211, ms213, ms215, ms216, ms217, and ms223. This scheme yielded a PCR product in 91-100% of the isolates and a high discriminatory index of 0.984 (CI 0.972–0.996).

### Comparison of typing methods

Subsequently we compared the adjusted MLVA9-Utrecht scheme with PFGE and MLST by typing 60 *P. aeruginosa* isolates from CF patients with the three methods. Typeability was 100% for MLST and MLVA9-Utrecht, but only 91.7% for PFGE as 5 isolates yielded, repeatedly, no banding patterns with this technique (Table 1). PFGE, MLVA9-Utrecht, and MLST distinguished 52, 45, and 36 types, respectively, which could be grouped in 33, 35, and 33 CCs. The DIs with 95% confidence intervals (CI) were comparable, although PFGE was slightly more discriminatory than MLST (Table 1). The three typing methods were highly congruent at the CC level with an adjusted Rand coefficient of 0.84 for PFGE vs. MLVA9-Utrecht, 0.91 for PFGE vs. MLST and 0.90 for MLST vs. MLVA9-Utrecht. Moreover, two strains that are of the same MT have a high probability of belonging to the same ST on the level of clonal clusters, as indicated by the Wallace coefficients (Table 2), which was highest between MLVA9-Utrecht and MLST (0.969).

**Table 1:** Typing characteristics of the genotyping methods for the 60 isolates typed with all 3 methods.

	PFGE	MLVA9-UTRECHT	MLST
Typeability	91.7%	100%	100%
Costs^a^	€5.78	€7.21	€121.60
Time^b^	5 days	2 days	7 days
Ease of interpretation	-	+	++
International comparison^c^	-	+	++
Discriminatory Index	0.998 [0.995–1.0]	0.982 [0.968–0.998]	0.963 [0.936–0.991]

a :cost per isolate tested, including materials, excluding labor and equipment depreciation since that is similar in all methods. MLST costs can be lower when not using outsourced sequencing.

b : Time can be shorter with MLST. In this study we outsourced sequencing that took extra time.

c : comparison with international data in database on http://pubmlst.org/paeruginosa/ for MLST and http://minisatellites.u-psud.fr/MLVAnet/ for MLVA.

**Table 2:** Wallace coefficients, indicating congruence between the different typing methods.

	MLVA9-UTRECHT	MLST	PFGE
MLVA9-UTRECHT	NA	0.969	0.917
MLST	0.845	NA	0.918
PFGE	0.793	0.910	NA

MLST-MLVA9-Utrecht comparison revealed that the previously identified high-prevalence STs among CF-patients, ST406 and ST497, were represented by MTs 27, 32, 52 and 238 and MTs 11 and 38, respectively.

Based on the high DI of MLVA9-Utrecht, the high congruence between this MLVA scheme and the other typing methods and the fact that MLVA is considerably cheaper than MLST, rapid to perform and allows data comparison with other datasets (Table 1), we selected MLVA9-Utrecht as the preferred typing method to determine the population structure of *P. aeruginosa* isolated from different epidemiological backgrounds in the Netherlands.

### Population biology of *P. aeruginosa* clinical isolates

All 363 isolates were typed with the adjusted 9 loci-MLVA scheme and 252 different MLVA9-Utrecht types could be discerned (typing data available in supplement Data S1). Typeability was 91% and ranged from 87% to 95% in the different patient groups (Table 3). In 22 and 10 isolates one or two loci could not be amplified, respectively, and these were assigned allele “99”. Of the loci that could not be amplified in all isolates, ms217, ms215 and ms77 could not be amplified in 12, 8 and 7 isolates, respectively. Ms127 was the only locus that could be amplified in all isolates. The genetic diversities in these four populations were similarly high, with an overall DI of 0.995 (CI 0.993–0.997) (Table 3).

**Table 3:** *P. aeruginosa* MLVA9-UTRECHT typing results of four different patient populations.

	group I	group IIa	group IIb	group II	group III	group IV	
	CF	ICU -I	ICU-II	ICU total	Hospital acquired Non-CF/non-ICU	Community acquired Non-CF/non-ICU	Total
source	respiratory	respiratory	respiratory	respiratory	non-respiratory	non-respiratory	diverse
# isolates (from # pat)	100 (90)	126 (97)	79 (64)	205 (161)	39 (38)	19 (19)	363 (308)
typeability	88%	93%	100%	95%	87%	89%	91%
# types	72	82	63	142	33	17	252
Index of diversity	0.984	0.981	0.991	0.991	0.989	0.988	0.995
CI	(0.971–0.996)	(0.97–0.992)	(0.983–0.999)	(0.987–0.996)	(0.977–1.0)	(0.969–1.0)	(0.993–0.997)
Prevalent types (≥5%)	MT27(11%) MT11(5%)	MT44 (10%) MT68 (6%)	MT161 (8%)	NA	MT255 (5%) MT261 (5%)	MT276 (11%) MT212 (11%)	NA

CF: cystic fibrosis patients, ICU: intensive care unit patients, MT: MLVA9-UTRECHT type,

#: number.

**Figure 1:**
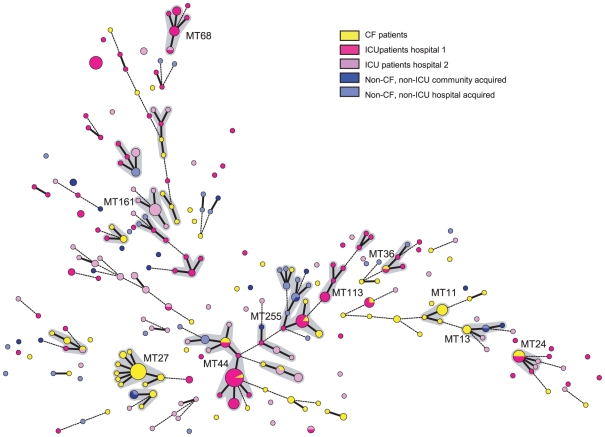
Minimum spanning tree of 363 *P. aeruginosa* isolates from different patient populations typed by MLVA9-UTRECHT. Circles represent MTs, the size of the circle is related to the number of isolates with that specific MT in this collection. Fat lines between the circles represent single locus variants (SLVs), differing only in one loci. Dotted lines represent double locus variants. Yellow color represents CF isolates, pink and purple are ICU respiratory isolates from two different hospitals, blue are non-CF non-ICU isolates (dark blue are “community acquired” isolates and light blue “hospital acquired”). Grey shading indicates clonal complexes.

The population structure of *P. aeruginosa* in this strain set based on MLVA9-Utrecht is characterized by a high level of host-specificity. Between 82% and 91% of MTs are unique for the different patient populations. Only 11 (4%) of the 252 MTs were detected in two different patient populations studied. These MTs represented 11% of the CF related types (group I), 6% of the ICU related types (group II), 9% of the non-ICU hospitalized patients (group III) and 18% of the community acquired types (group IV), respectively (table 4). The MTs found in groups III and IV (hospitalized, non-ICU patients and patients with community acquired isolates) were not found in groups I (CF patients) or II (ICU patients). When comparing MTs from the ICU populations in both hospitals, most MTs appeared to be ICU-specific (fig. 1/table 4). Only three (2%) of 142 MTs were detected in samples from patients in both ICUs indicating specific clustering in both location and patient group. The DIs of CF group and ICU-1 group appeared significantly lower than what would have been expected in case of random distribution of MTs (table 5). Furthermore, calculation of expected unique and shared types between the five groups in case of random distribution revealed that the observed numbers of shared types between all the different groups was significantly lower than what would have been expected, except between group III and IV (table 4). This proves non-random clustering of MTs and the presence of patient group-specific types.

**Table 4:** Numbers (%) of shared and unique MLVA9-UTRECHT types (MTs) in the four groups of clinical sources compared to the numbers of expected values based on 100.000 permutations (median, range and 95% confidence interval (CI)) when assuming random distribution of types.

			MTs shared (percentage of total MTs) [95% CI]				
		Unique	CF	ICU-I	ICU-2	HA	CA
Source			group I	group IIa	group IIb	group III	group IV
CF	Observed	64 (89%)		6 (8%)^a^	2 (3%) a	0 a	0 a
	Expected	58 [50–66]		18 [13]–[23]	13 [8]–[18]	8 [4]–[12]	4 [1]–[7]
ICU-1	Observed	72 (88%)	6 (7%) a		3 (4%) a	0 a	0 a
	Expected	74 [66–82]	18 [12]–[23]		15 [10]–[20]	9 [5]–[13]	5 [2]–[8]
ICU-2	Observed	58 (92%) b	2 (3%) a	3 (5%) a		0 a	0 a
	Expected	46 [38–53]	13 [8]–[18]	15 [10]–[20]		7 [3]–[11]	3 [1]–[7]
HA	Observed	30 (91%) b	0 a	0 a	0 a		3 (9%)
	Expected	23 [17]–[28]	8 [4]–[12]	9 [5]–[13]	7 [3]–[11]		2 [0–5]
CA	Observed	14 (82%)	0 a	0 a	0 a	3 (18%)	
	Expected	10 [6]–[14]	4 [1]–[7]	5 [2]–[8]	3 [1]–[7]	2 [0–5]	

CF: cystic fibrosis patients, ICU: intensive care unit patients, HA: non-CF, non-ICU patients with hospital acquired *P. aeruginosa*, CA: non-CF, non-ICU patients with community acquired *P. aeruginosa*.

a : value lower than expected within 95% CI range, i.e. less overlap of types between sources than in the case of random distribution of types.

b : more unique genotypes per source than expected, i.e. high level of source-specificity rather than random distribution.

**Table 5:** Expected Indices of Diversity (DI) and 95% confidence intervals based on 100.000 permutations based on random distribution of genotypes compared to observed DI.

	CF	ICU-1	ICU-2	HA	CA
Observed DI	0.984 *	0.981*	0.991	0.989	0.988
expected DI	0.995	0.995	0.995	0.996	1.0
[95% CI]	[0.991–0.998]	[0.992–0.997]	[0.991–0.998]	[0.987–1.0]	[0.982–1.0]

*not within the 95% confidence interval (CI) range; i.e. diversity in that specific group is lower than would be expected on random distribution of types.

The 252 MTs could be grouped in 22 CCs, defined as clusters of three or more types that share at least 8 out of 9 loci (fig. 1). The minimum spanning tree revealed that specific clustering in both location and patient group did not result in grouping of isolates from a single patient population in one genetic lineage or genetic subpopulation. In contrast, isolates belonging to a single patient population are scattered over the minimum spanning tree. In agreement with the observed host-specificity, only three CCs contained isolates from four of the five populations studied. These three CCs (CC44, CC255 and CC13) contain CF isolates that were also typed by MLST allowing comparison with other isolates in the MLST database [19]. These “mixed” CCs, detected in each patient population, are closely related to *P. aeruginosa* clones that had been detected up to 7 countries on 4 continents.

Three CCs contained isolates from CF patients only. Two of these, CC27 and CC11, contained the two previously reported high prevalent genotypes in CF-patients, ST406 and ST497, represented in this study by MLVA9-Utrecht types MT 27, 32, 52 and 238 (CC27) and MT 11 and 38 (CC11), respectively. This means that these two high prevalent CF clones are exclusively found in CF patients (fig. 1).

## Discussion

Using a simplified MLVA scheme for genotyping we have demonstrated that the population structure of *P. aeruginosa* isolates is highly diverse and population specific. This implies that most clones specific for CF patients, including the highly prevalent Dutch clones MT27, 32, 52, 238 (ST406) and MT 11, 38 (ST497), are genetically distinct from clones from non-CF patients. The high prevalence of these clones in CF patients, therefore, is unlikely to result from transmission of particular dominant clones from the non-CF reservoir. Moreover, ICU-wards from different hospitals appeared to have location specific *P. aeruginosa* populations.

MLVA9-Utrecht revealed that the *P. aeruginosa* population in the different clinical settings is highly diverse with a DI of 0.995 with no difference in diversity between hospital acquired and community acquired strains. This corroborates with previous findings in ICU patients [20], [21].

Studies in the last decade have proposed different types of *P. aeruginosa* population structures, ranging from panmictic in the early nineties [22], [23] to more clonal in 2007 [24]. The latest reports however, summarized by Pirnay in 2009, point towards a nonclonal epidemic population structure, with no distinction between clinical or environmental isolates [25]. In particular, the lack of distinction in genotype, function and chemotaxonomy between clinical and environmental *P. aeruginosa* isolates has been reported by different research groups [26], [27]. Based on FAFLP, gene sequencing and virulence gene profiling Pirnay *et al* described that strains which clustered in the same clonal complexes could have been isolated from inanimate environments, animals and humans, sometimes separated by thousands of miles. They concluded that there was no correlation between the clonal complexes and geographical origin or habitat. We also found that the three clonal complexes that were present in the four epidemiological backgrounds had been detected previously in up to seven other countries on four continents, indicating their global presence [28].

However, in contrast to previous research that suggested no correlation between *P. aeruginosa* clones and diseases or environmental habitats [29], we found genotypes to be highly specific for the different patient groups with only a relatively small number of clones distributed across patient population boundaries. However, since the MLVA database [28] does not provide data on the source of the isolate we cannot elaborate on the association between these types and epidemiological background. Our findings of high specificity of different sets of genotypes, not only in the various patient groups but also between ICUs in the different hospitals, are remarkable. Thus, discordant to the proposed consensus of a non-clonal epidemic population structure with some dominant clonal complexes, which are just as versatile in their habitat and geographic origin as the whole *P. aeruginosa* population, we found that both patient population and geographical origin appeared to be correlated to the prevalence of certain genotypes and that transmission of *P. aeruginosa* clones between ICUs, hospital wards and CF patients is rare.

The limited overlap between isolates from CF and non-CF patients also fails to support findings reported by Lanotte *et al*, who described, based on random amplification of polymorphic DNA (RAPD), a non-random distribution of isolates but with a subpopulation of isolates originating from patients with lung disease, both CF and non-CF. This could result from low discriminatory power of RAPD.

Pirnay *et al* also concluded that, based on typing of 328 unrelated isolates including 43 CF isolates, all CF isolates clustered into a “core lineage” that is predominant in both disease and environmental habitats across the world. Consequently, CF isolates belonging to the so-called “successful core lineage” are ubiquitous in the natural environment and are, therefore, more likely to infect CF patients. We failed to confirm such a level of “relatedness” in our populations, as CF isolates, as well as the ICU isolates and other clinical isolates were dispersed over the entire minimum spanning tree. Moreover, the two most successful CF clones in our country were not detected in other patient populations and they are not genotypically closely related to non-CF isolates. This suggests no common evolutionary background of *P. aeruginosa* isolates from CF patients nor of *P. aeruginosa* isolates from the other analyzed patient groups. Our findings are more in line with the observation that the Australian Epidemic strains I and III (AESI and AESIII) could not be isolated from the environment [30], [31]. These findings suggest selection of multiple specific clones with a distinct evolutionary background that are better equipped to adapt to and survive in the specific conditions in the CF lung. This also indicates that *P. aeruginosa* from many different lineages can adapt to all kinds of niches. This concurs with data from Pirnay *et al*, who found that a *P. aeruginosa* community in a Belgian river contained members of nearly all successful clonal complexes and was almost as diverse as the global population, represented by 73 clinical and environmental isolates from a previous study [32].

The strength of our study is the large and well-defined collection of isolates and the ability of MLVA9-Utrecht to show, highly reproducible, genotypic relatedness, with the possibility of comparing the genotypes to results contained in international databases via the internet, and that can be performed under point-of-care conditions. One should be aware that the MTs assigned in this study only refer to the MLVA9-Utrecht scheme. We did not include isolates from the environment in our study, shown to contain similar genotypes as clinical isolates in other studies, which may change our findings of specificity.

We conclude that the population structure of *P. aeruginosa* from different patient populations is highly diverse and characterized by high-level host-specificity and by the presence of many unique and only a limited number of more prevalent genotypes. The two genotypes (MT27/ST406 and MT11/ST497), frequently found in the Dutch CF patients, appear to be unique to CF patients and are not found in other clinical patients. Further studies are needed to elucidate the specific adaptations and survival strategies that these strains have adopted to survive in this special niche.

## Supporting Information

Data S1 MLVA data file.
(0.08 MB XLS)
